# Emergency laparoscopic repair of an incarcerated obturator hernia, a case report

**DOI:** 10.1016/j.ijscr.2020.06.063

**Published:** 2020-06-20

**Authors:** Francis Zheng Yi Yee, Aik Yong Chok, Ting Hway Wong

**Affiliations:** a Department of General Surgery, Singapore General Hospital, Outram Road, 169608, Singapore; b Department of Colorectal Surgery, Singapore General Hospital, Singapore; c Duke-NUS Medical School, Singapore

**Keywords:** Obturator hernia, Laparoscopic repair, Case report

## Abstract

Obturator hernias are rare hernias with high morbidity and mortality if not diagnosed and treated promptly. Various minimally invasive techniques have been reported in literature. Here we describe a novel technique of repairing an obturator hernia complicated by small bowel incarceration and obstruction, via a laparoscopic trans-abdominal approach.

## Highlights

• Obturator hernias have high morbidity and mortality which require emergency treatment.
• These hernias are usually treated via open surgery despite the advancement of laparoscopic surgery.
• We describe and demonstrate a repair method via minimally invasive surgery.
• Laparoscopic repair is feasible in the emergency setting.

## 1 Case report

We present a case in line with the SCARE Guidelines [1]. A 76-year-old Chinese lady was recently diagnosed with Antineutrophil cytoplasmic antibody (ANCA)-associated vasculitis, and started on high-dose intravenous pulsed methylprednisolone during her admission to our academic medical centre. During her inpatient stay, she developed small bowel obstruction, with the Computed Tomography (CT) scan demonstrating the presence of a left incarcerated obturator hernia. She underwent emergency laparoscopic repair of left obturator hernia and primary repair of small bowel defect by two consultant-level surgeons.

During reduction of the incarcerated small bowel loop, bowel perforation was encountered and the defect was controlled with a bowel grasper by the assistant. Subsequently the hernia sac was inverted and closed with an endoloop. The base was reinforced with a figure-of-eight suture and the excess sac was excised. The bowel wall defect was primarily repaired through a small midline incision.

## 2 Discussion

Obturator hernias are rare, having an incidence of 0.073%, and are usually diagnosed when presenting with bowel obstruction or perforation [2]. The incidence is higher in females, especially those who are emaciated and elderly. Obturator hernias are associated with the highest morbidity rates amongst all abdominal wall hernias, between 13–40% [3], this is contributed to by high rates of strangulation and subsequent bowel wall necrosis due the small hernial defect. These hernias are usually diagnosed either preoperatively with a CT of the abdomen and pelvis or intraoperatively during an exploratory laparotomy or laparoscopy.

The various techniques that have been described for open repair include primary suture repair, fascial and muscular flaps, omental plugs, simple ligation of the sac base, mobilization of the round ligament for coverage, and mesh repair [4,5].

More frequently adopted in the elective setting, laparoscopic obturator hernia repair can be performed via the totally extraperitoneal (TEP) and transabdominal preperitoneal (TAPP) approaches. In patients with intestinal obstruction secondary to strangulation or incarceration, the laparoscopic transabdominal approach enables direct visualization of bowel, and reduction and resection if necessary.

In this report, we describe and demonstrate a simple and effective laparoscopic transabdominal repair in the emergency setting, without the use of a mesh in view of intraperitoneal contamination.

## 3 Conclusion

Laparoscopic transabdominal repair of incarcerated obturator hernia is feasible in the emergency setting.

## Declaration of Competing Interest

None.

## Sources of funding

None.

## Ethical approval

Exempt from ethical approval as this is not a research study.

## Consent

Written consent was obtained from the patient for publication. Identifiable patient information has been removed.

## Author contribution

Francis Yee: Writing - Original Draft, Visualization.

Chok Aik Yong: Writing - Review & Editing, Visualization.

Wong Ting Hway: Writing - Review & Editing, Visualization.

## Registration of research studies

N/A.

## Guarantor

N/A.

## Provenance and peer review

Not commissioned, externally peer-reviewed.
